# A modified Holzapfel-Ogden law for a residually stressed finite strain model of the human left ventricle in diastole

**DOI:** 10.1007/s10237-013-0488-x

**Published:** 2013-04-23

**Authors:** H. M. Wang, X. Y. Luo, H. Gao, R. W. Ogden, B. E. Griffith, C. Berry, T. J. Wang

**Affiliations:** 1 School of Civil Engineering, Xinjiang University, Xinjiang, China; 2 School of Mathematics and Statistics, University of Glasgow, Glasgow, UK; 3 Leon H. Charney Division of Cardiology, Department of Medicine, New York University School of Medicine, New York, NY USA; 4 Institute of Cardiovascular and Medical Sciences, University of Glasgow, Glasgow, UK; 5 SV Lab, School of Aerospace Engineering, Xi’an Jiaotong University, Xi’an, China

**Keywords:** Residual stress, Left ventricle, Finite strain, Finite stress

## Abstract

In this work, we introduce a modified Holzapfel-Ogden hyperelastic constitutive model for ventricular myocardium that accounts for residual stresses, and we investigate the effects of residual stresses in diastole using a magnetic resonance imaging–derived model of the human left ventricle (LV). We adopt an invariant-based constitutive modelling approach and treat the left ventricular myocardium as a non-homogeneous, fibre-reinforced, incompressible material. Because in vivo images provide the configuration of the LV in a loaded state even in diastole, an inverse analysis is used to determine the corresponding unloaded reference configuration. The residual stress in this unloaded state is estimated by two different methods. One is based on three-dimensional strain measurements in a local region of the canine LV, and the other uses the opening angle method for a cylindrical tube. We find that including residual stress in the model changes the stress distributions across the myocardium and that whereas both methods yield qualitatively similar changes, there are quantitative differences between the two approaches. Although the effects of residual stresses are relatively small in diastole, the model can be extended to explore the full impact of residual stress on LV mechanical behaviour for the whole cardiac cycle as more experimental data become available. In addition, although not considered here, residual stresses may also play a larger role in models that account for tissue growth and remodelling.

## Introduction

Even in the absence of externally applied loads, soft tissues generally are not stress-free. The stresses that remain after all external loads have been removed are termed *residual stresses*. Residual stresses are present in a large variety of biological tissues and result from tissue growth and remodelling occurring over the life span of the tissue (Bovendeerd et al. 1994).

Fung (1993) and Vaishnav and Vossoughi (1987) appear to have been the first to consider residual stresses in soft tissues. They found that when a length of artery is excised from a body, the artery contracts. Thus, in vivo, arteries are stretched (i.e. subject to a large axial deformation) and tethered (i.e. held in place) by the surrounding tissue. However, although an excised artery is not subject to any axial load or to any traction on its inner and outer surfaces, it is not unstressed; rather, there remains a residual stress distribution across the arterial wall. The existence of these residual stresses may be demonstrated by the so-called *opening angle experiment*, first proposed by Chuong and Fung (1986), in which a short length of artery in the form of a ring is cut radially. The ring springs open to form an open sector, thus indicating the presence of a compressive circumferential stress in the inner part of the wall of the ring and a tensile circumferential stress in the outer part. The studies by Han and Fung (1996) and by Liu and Fung (1989) suggest that most of the residual stress is released by a single cut. In contrast, Vossoughi et al. (1993), Greenwald et al. (1997), and Schulze-Bauer et al. (2002) have shown that a single cut is not sufficient to relieve all the residual stress in arterial walls. Residual stresses have an important influence on the mechanical response of the artery under physiological conditions. It is believed that residual stress tends to reduce the stress concentration at the inner arterial wall (Chuong and Fung 1986), and it has been speculated that residual stresses are distributed so that the stress distributions across the arterial wall layers are more uniform at physiological pressures (Rachev and Greenwald 2003).

Finite strain and stress analyses of the left ventricular wall can further our understanding of the heart in health and disease. Despite extensive studies of residual stress in arteries, there have been relatively few studies on residual stress and strain in the myocardium. Residual stresses could have an influence on the dynamics of the left ventricle (LV) and on transmural stress and strain distributions. To date, however, most ventricular mechanics models have assumed that the unloaded configuration is a stress-free configuration (Mirsky 1973; Demiray 1976a, b; Bovendeerd et al. 1994; Wang et al. 2013). Indeed, it is an ongoing challenge to recover residual stress in myocardial tissues both theoretically and experimentally.

Omens and Fung (1990) studied residual strains in rat LV by measuring the opening angles of equatorial LV slices. They discovered that equatorial rings opened into arcs with a mean opening angle of about $45^\circ$. Once ischaemic contracture had set in, they observed a continual increase in the opening angle, up to approximately $180^\circ$, that was associated with a dramatic increase in specimen stiffness. Residual strains were found to be negative (compressive) in the endocardium and positive (tensile) in the epicardium. Rodriguez et al. (1993) studied the effects of residual stress on the transmural sarcomere length (SL) distributions in the equatorial region of the rat LV. Upon comparing the distributions of SL between the unloaded but residually stressed state and the stress-free state, they found that the SL was uniform across the wall in the stress-free state; however, in intact tissue, there was a significant decrease in SL from epicardium to endocardium. This gradient is believed to offset the opposing gradient in sarcomere extension during filling, thus leading to a more uniform transmural distribution of SL at end diastole and hence more uniform development of systolic force. Summerour et al. (1998) used the opening angle method to estimate residual strains in equatorial slices of normal and ischaemic rat LV. They did not observe obvious differences in the residual stress distributions between normal and ischaemic myocardium. Costa et al. (1997) performed in vitro experiments using biplane radiography in which columns of beads implanted in the mid-anterior free wall of the canine LV determined transmural distributions of the three-dimensional residual strains. To date, no experimental studies have been reported on the residual stress and strain distributions in human myocardium.

Using analytical and numerical modelling, Guccione et al. (1991) studied the passive mechanics of the canine LV using a thick-walled cylindrical LV model in which the myocardium is treated as an incompressible hyperelastic material. They further assumed that the LV is transversely isotropic and may be described by a four-parameter Fung-type model and that the residual stress is isotropic. They looked at the effects of residual strain and stress on the circumferential stress distributions when using opening angles. Nevo and Lanir (1994) carried out a similar study on the influence of residual strain on the diastolic function of the LV using a structural model. Nash (1998) assumed an initial strain field based on the experimental data of Omens and Fung (1990) and used the finite element method and the *pole-zero* constitutive law (Hunter et al. 1997) to develop an anatomically accurate mathematical model of canine LV. Their computational results, which covered the whole cardiac cycle, showed that the effects of residual strain in the LV are small. Guccione et al. (2001) also used the finite element method to estimate residual stresses during ventricular volume reduction surgery. When they evaluated the impact of the residual stress on ventricular function, they also found that the effects of these stresses are small. Taber (1991) simulated the beating left ventricle using a nonlinear laminated thick-shell model with residual strains and discovered that the residual strains in the LV significantly affect the peak fibre stress and transmural stress gradients near the beginning of systole. Because of the transmural changes in fibre orientation in the LV, these effects are not as large as they are in arteries.

Although most studies on the role of residual stresses in the LV myocardium suggest that the effects of these stresses are smaller than those in arteries, mathematically, it is appropriate to evaluate the actual stress with residual stress contribution included. In addition, accounting for residual stresses may be important in models that describe tissue growth and remodelling because the growth and remodelling processes may themselves act to set up and to maintain residual stresses. Recently, Holzapfel and Ogden (2009) proposed a structure-based constitutive model of ventricular myocardium that accounts for the locally orthotropic tissue microstructure by expressing the strain-energy function using fibre-based material invariants. In the incompressible case, their strain-energy function has eight material parameters with relatively clear physical meanings. Moreover, this model satisfies convexity and strong ellipticity properties that are important both mathematically and physically. In this work, we adopt the approach of Shams et al. (2011), who describe a general invariant-based method for incorporating residual stresses into hyperelastic material models, to extend the Holzapfel-Ogden constitutive law to account for residual stresses.

In previous work, we developed a three-dimensional computational model of the human LV that is derived from non-invasive magnetic resonance imaging (MRI) data (Wang et al. 2013). This anatomically realistic model has a rule-based fibre structure and, in our earlier study, used the original structure-based constitutive model of Holzapfel and Ogden (2009) (i.e. without residual stresses). In this work, we use the method introduced by Shams et al. (2011) to modify the Holzapfel-Ogden law so that the residual stresses are taken into account. This constitutive model, along with our MRI-based anatomic model, is then used to investigate the influence of residual stress on the mechanical behaviour of the LV in diastole.

## Constitutive laws for the passive myocardium 

### Constitutive law based on stress-free configuration 

**Fig. 1:**
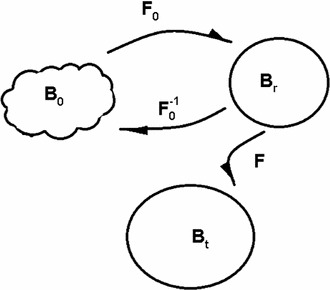
Schematic of the relationships between the stress-free reference configuration $B_0$, the residually stressed configuration $B_r$, and the deformed configuration $B_t$, showing the connecting deformation gradients $\mathbf{F}_0, \mathbf{F}^{-1}_0$, and $\mathbf{F}$

Consider a continuum body with stress-free configuration $B_0 \subset \mathbb{R }^3$, residually stressed and unloaded configuration $B_r \subset \mathbb{R }^3$, and current configuration $B_t \subset \mathbb{R }^3$, as shown in Fig. 1. These configurations are related by a time-dependent mapping $\varvec{\chi }_0: B_0 \times [0, T] \rightarrow B_t$, and $\varvec{\chi }: B_r \times [0, T] \rightarrow B_t$. Let $\mathbf{X}_0$ denote coordinates in the configuration $B_0, \mathbf{X}$ denote coordinates in the configuration $B_r$, and $\mathbf{x}$ denote coordinates in the current configuration $B_t$. If the residual stress is zero, then $B_r=B_0$, $\varvec{\chi }=\varvec{\chi }_0$, and the deformation gradient tensor associated with the motion $\mathbf{x}=\varvec{\chi }(\mathbf{X},t)$ is $\mathbf{F}=\partial \varvec{\chi }/\partial \mathbf{X}=\partial \varvec{\chi }_0/\partial \mathbf{X}_0$.

We treat the LV myocardium as a non-homogeneous, nonlinear, incompressible, and hyperelastic material. The constitutive model is described in terms of invariants of the right Cauchy–Green deformation tensor $\mathbf{C}=\mathbf{F}^T\mathbf{F}$,

1 $$\begin{aligned} I_{1} = \text{ tr }(\mathbf{C}), I_{2}=\frac{1}{2}[{I_1}^2 - \text{ tr } (\mathbf{C}^2)], I_{3} = \det (\mathbf{C}), \end{aligned}$$

along with other invariants that are defined below. Because we model the ventricular myocardium as an incompressible material, $J=\det \mathbf{F}=1$, and, hence, $I_{3}=1$.

**Fig. 2:**
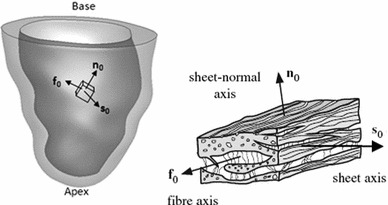
Local coordinate axes of the left ventricle, in which $(\mathbf{f}_0, \mathbf{s}_0, \mathbf{n}_0)$ are the fibre, sheet, and sheet–normal axes, as detailed in the *right insert*  (taken from Holzapfel and Ogden (2009)) and described in the text

The orthotropic structure of the LV myocardium is described in the model in terms of the fibre axis $\mathbf{f}_0$, the sheet (cross-fibre) axis $\mathbf{s}_0$, and the sheet–normal axis $\mathbf{n}_0 = \mathbf{f}_0 \times \mathbf{s}_0$, as shown in Fig. 2. Using these material axes, additional quasi-invariants can be defined to characterize the material response in these preferred directions (Spencer 1984). In this work, we follow Holzapfel and Ogden (2009) by introducing the fibre, sheet, and fibre–sheet invariants,

2 $$\begin{aligned} I_{4\text{ f }}=\mathbf{f}_0 \cdot (\mathbf{C}\mathbf{f}_0), I_{4\text{ s }}=\mathbf{s}_0 \cdot (\mathbf{Cs}_0), I_{4\text{ fs }}=\mathbf{f}_0 \cdot (\mathbf{Cs}_0), \end{aligned}$$

and write the structure-based strain-energy function $W = W(I_1,I_{4\text{ f }},I_{4\text{ s }},I_{4\text{ fs }})$ as

3 $$\begin{aligned} W&= \frac{a}{2b} \exp [b(I_1-3)] \nonumber \\&\quad + \,\,\sum _{i=\text{ f },\text{ s }} \frac{a_i}{2b_i}\{\exp [b_i(I_{4i}-1)^2]-1\} \nonumber \\&\quad + \,\,\frac{a_\text{ fs }}{2b_\text{ fs }}\{\exp [b_\text{ fs }(I_{4\text{ fs }})^2]-1\}, \end{aligned}$$

in which $a,\,b,\,a_i$, and $b_i$ ($i=\text{ f },\,\text{ s }$, and $\text{ fs }$) are eight non-negative material parameters. The term $I_{4\text{ fs }}$ plays the same role as the quantity denoted $I_{8\text{ fs }}$ by Holzapfel and Ogden (Holzapfel and Ogden 2009). This is because a quantity denoted $I_8$ will be used below in defining the residual stress state. The first term in (3) is a Fung-type expression that corresponds to the contributions to the strain-energy function of an isotropic ground matrix material. The remaining terms correspond to the contributions of the cardiac myocytes and families of collagen fibres embedded within the tissue. Because we assume that the fibres support only extension and not compression, the terms involving $I_{4\text{ i }}$ for $i=\text{ f }$ and $\text{ s }$ are included in the total energy only if $I_{4\text{ i }} > 1$.

For an incompressible material, the Cauchy stress tensor can be derived from (3):

4 $$\begin{aligned} \varvec{\sigma }&= -p\mathbb{I } + \mathbf{F} \sum _{i=1,4\text{ f },4\text{ s },4\text{ fs }} \frac{\partial W}{\partial I_i} {\frac{\partial I_i}{\partial \mathbf{F}}} \nonumber \\&= -p\mathbb{I } + a\exp [b(I_1-3)]\mathbf{B} \nonumber \\&\quad +2a_\text{ f }(I_{4\text{ f }}-1)\exp [b_\text{ f }(I_{4\text{ f }}-1)^2] \mathbf{f} \otimes \mathbf{f} \nonumber \\&\quad +2a_\text{ s }(I_{4\text{ s }}-1)\exp [b_\text{ s }(I_{4\text{ s }}-1)^2] \mathbf{s} \otimes \mathbf{s} \nonumber \\&\quad + a_\text{ fs }I_{4\text{ fs }}\exp [b_\text{ fs }(I_{4\text{ fs }})^2](\mathbf{f} \otimes \mathbf{s} + \mathbf{s} \otimes \mathbf{f}), \end{aligned}$$

in which $p$ is a Lagrange multiplier introduced to enforce incompressibility, $\mathbb{I }$ is the identity tensor, $\mathbf{B}=\mathbf{F}\mathbf{F}^T$ is the left Cauchy–Green deformation tensor, and $\mathbf{f}=\mathbf{F}\mathbf{f}_0$ and $\mathbf{s}=\mathbf{F}\mathbf{s}_0$ are the fibre and sheet axes in the current (deformed) configuration, respectively.

### Constitutive law for the residually stressed myocardium

To describe the constitutive law for the residually stressed myocardium, we choose $B_r$ as the reference configuration. Any subsequent deformation of the body is measured from ${B_r}$, and we assume that there is an initial Cauchy stress $\varvec{\tau }$ in this configuration.^1^ If the traction on the boundary $\partial B_r$ of $B_r$ vanishes, then $\varvec{\tau }$ is referred to as the residual stress, which is necessarily non-uniform and symmetric and satisfies the equilibrium equation (Johnson and Hoger 1995; Shams et al. 2011):

5 $$\begin{aligned} \text{ Div }(\varvec{\tau }) = 0. \end{aligned}$$

For the myocardium, we propose that the strain-energy function $W$ and Cauchy stress $\varvec{\sigma }$ depend on the residual stress $\varvec{\tau }$ as well as $I_i$ ($i=1,\,4\text{ f },\,4\text{ s }$, $4\text{ fs }$), namely

6 $$\begin{aligned} W&= W(\mathbf {F},\varvec{\tau }),\end{aligned}$$

7 $$\begin{aligned} \varvec{\sigma }&= \mathbf{F} \frac{\partial W}{\partial \mathbf{F}}(\mathbf{F}, \varvec{\tau }) -p\mathbb{I }. \end{aligned}$$

Following Shams et al. (2011), we introduce the additional invariants

8 $$\begin{aligned} I_6&= \text{ tr }(\varvec{\tau }\mathbf{C}),\,I_7 = \text{ tr }(\varvec{\tau }\mathbf{C}^2), \nonumber \\ I_8&= \text{ tr }(\varvec{\tau }^2 \mathbf{C}), \text{ and } I_9 = \text{ tr }(\varvec{\tau }^2 \mathbf{C}^2) \end{aligned}$$

that depend on both $\mathbf{C}$ and $\varvec{\tau }$. For simplicity, we do not include invariants that involve $\varvec{\tau },\,\mathbf{C}$, and the fibre directions that are included in the general theory by Ogden and Singh (2011). The Cauchy stress tensor is now (Shams et al. 2011)

9 $$\begin{aligned} \varvec{\sigma }&= -p\mathbb{I } + \mathbf{F} \sum _{i} \frac{\partial W}{\partial I_i} {\frac{\partial I_i}{\partial \mathbf{F}}} \nonumber \\&= -p\mathbb{I } + a\exp [b(I_1-3)]\mathbf{B} \nonumber \\&\quad +2a_\text{ f }(I_{4\text{ f }}-1)\exp [b_\text{ f }(I_{4\text{ f }}-1)^2] \mathbf{f} \otimes \mathbf{f} \nonumber \\&\quad +2a_\text{ s }(I_{4\text{ s }}-1)\exp [b_\text{ s }(I_{4\text{ s }}-1)^2] \mathbf{s} \otimes \mathbf{s} \nonumber \\&\quad +a_\text{ fs }I_{4\text{ fs }}\exp [b_\text{ fs }(I_{4\text{ fs }})^2](\mathbf{f} \otimes \mathbf{s} + \mathbf{s} \otimes \mathbf{f})\nonumber \\&\quad +2W_6\varvec{\Sigma } +2W_7(\varvec{\Sigma } \mathbf{B}+\mathbf{B}\varvec{\Sigma })+2W_8\varvec{\Sigma } \mathbf{B}^{-1}\varvec{\Sigma } \nonumber \\&\quad +2W_9(\varvec{\Sigma } \mathbf{B}^{-1}\varvec{\Sigma } \mathbf{B}+ \mathbf{B}\varvec{\Sigma } \mathbf{B}^{-1}\varvec{\Sigma }), \end{aligned}$$

in which $W_i = \frac{\partial W}{\partial I_i} (i=1,4\text{ f },4\text{ s },4\text{ fs },6,7,8,9),$ and $\varvec{\Sigma } = \mathbf{F}\varvec{\tau }\mathbf{F}^T$.

Notice that in the unloaded but residually stressed configuration $B_r,\,\mathbf{F} =\mathbb{I }$, and

$$\begin{aligned}&\mathbf{B} =\mathbb{I }, I_1=3, I_{4\text{ f }}=I_{4\text{ s }}=1, I_{4\text{ fs }}=0,\\&\varvec{\Sigma }=\varvec{\tau }, I_6=I_7=\text{ tr }(\varvec{\tau }), I_8=I_9=\text{ tr }(\varvec{\tau }^2), \end{aligned}$$

and hence $\varvec{\sigma }$ in (7) reduces to $\varvec{\tau }$, i.e.,

10 $$\begin{aligned} \varvec{\tau }=\frac{\partial W}{\partial \mathbf{F}}(\mathbb{I },\varvec{\tau })-p^{(r)}\mathbb{I }, \end{aligned}$$

in which $p^{(r)}$ is the value of $p$ in $B_r$. Consequently, (9) becomes

11 $$\begin{aligned} \varvec{\tau }=(a-p^{(r)})\mathbb{I }+2(W_6+2W_7)\varvec{\tau }+2(W_8+2W_9)\varvec{\tau }^2, \end{aligned}$$

where all $W_i$ are evaluated at the unloaded configuration, $B_r$. This indicates that

12 $$\begin{aligned} 2W_1\!=\!p^{(r)}= a,\quad 2(W_6+2W_7)\!=\!1,\quad W_8\!+\!2W_9\!=\!0,\quad \end{aligned}$$

in $$B_r$$.

Notice that (11) tells us nothing about the specific form of $\varvec{\tau }$, which could arise through various routes, including residual strain with respect to the zero-stress configuration, and which could possibly be determined by a constitutive law that is different from (3).

In the *simple approach* to including residual stresses, we consider small strains, so that $\mathbf{B} \approx \mathbb{I }$. Hence, $\varvec{\Sigma } \mathbf{B}\approx \varvec{\Sigma }$ and $\mathbf{B}\varvec{\Sigma }\approx \varvec{\Sigma }$ in (9). Consequently, we include only the $W_6$ term. For simplicity, we also assume that $W_6$ is constant. Then, for consistency with (12)$_2,\,W_6=1/2$. Under these assumptions along with (12), the modified strain-energy function $W$ becomes

13 $$\begin{aligned} W&= W(I_1, I_{4\text{ f }}, I_{4\text{ s }}, I_{4\text{ fs }}, I_6)\nonumber \\&= \frac{a}{2b}\exp [b(I_1-3)] \nonumber \\&\quad +\sum _{i=\text{ f },\text{ s }} {\frac{a_i}{2b_i}\{\exp [b_i(I_{4i}-1)^2]-1\}} \nonumber \\&\quad +\frac{a_\text{ fs }}{2b_\text{ fs }} \{\exp [b_\text{ fs }(I_{4\text{ fs }})^2]-1\} + \frac{1}{2}I_6. \end{aligned}$$

In the *extended approach*, we also specifically include the term $2W_7 (\varvec{\Sigma } \mathbf{B}+\mathbf{B}\varvec{\Sigma })$ in (9), assuming $W_7$ is constant. Similar to the simple approach, consistency with (12)$_2$ requires $W_6=1/4$ and $W_7=1/8$. The strain-energy function $W$ is

14 $$\begin{aligned} W&= W(I_1,I_{4\text{ f }}, I_{4\text{ s }}, I_{4\text{ fs }}, I_6, I_7)\nonumber \\&= \frac{a}{2b}\exp [b(I_1-3)] \nonumber \\&\quad + \sum _{i=\text{ f },\text{ s }} {\frac{a_i}{2b_i}\{\exp [b_i(I_{4i}-1)^2]-1\}} \nonumber \\&\quad + \frac{a_\text{ fs }}{2b_\text{ fs }} \{\exp [b_\text{ fs }(I_{4\text{ fs }})^2]-1\} + \frac{1}{4}I_6 + \frac{1}{8}I_7. \end{aligned}$$

We remark that adding the residual stress in these approaches does not introduce any extra material parameters. The next level of sophistication is to consider all terms, including $I_8$ and $I_9$, in (11); however, as we shall see below, this becomes unnecessary as the residual stress is small. For the simple approach, the Cauchy stress tensor is:

15 $$\begin{aligned} \varvec{\sigma }&= -p\mathbb{I } + a\exp [b(I_1-3)]\mathbf{B}\nonumber \\&\quad + 2a_\text{ f }(I_{4\text{ f }}-1) \exp [b_\text{ f }(I_{4\text{ f }}-1)^2] \mathbf{f}\otimes \mathbf{f} \nonumber \\&\quad + 2a_\text{ s }(I_{4\text{ s }}-1) \exp [b_\text{ s }(I_{4\text{ s }}-1)^2] \mathbf{s} \otimes \mathbf{s} \nonumber \\&\quad + a_\text{ fs }I_{4\text{ fs }} \exp [b_\text{ fs }(I_{4\text{ fs }})^2] (\mathbf{f} \otimes \mathbf{s} + \mathbf{s} \otimes \mathbf{f}) + \varvec{\Sigma }. \end{aligned}$$

For the extended approach, the Cauchy stress tensor is:

16 $$\begin{aligned} \varvec{\sigma }&= -p\mathbb{I } + a\exp [b(I_3)]\mathbf{B}\nonumber \\&\quad + 2a_\text{ f }(I_{4\text{ f }}-1) \exp [b_\text{ f }(I_{4\text{ f }}-1)^2] \mathbf{f}\otimes \mathbf{f} \nonumber \\&\quad + 2a_\text{ s }(I_{4\text{ s }}-1) \exp [b_\text{ s }(I_{4\text{ s }}-1)^2] \mathbf{s} \otimes \mathbf{s} \nonumber \\&\quad + a_\text{ fs }I_{4\text{ fs }} \exp [b_\text{ fs }(I_{4\text{ fs }})^2] (\mathbf{f} \otimes \mathbf{s} + \mathbf{s} \otimes \mathbf{f}) \nonumber \\&\quad + \frac{1}{2}\varvec{\Sigma } + \frac{1}{4}(\varvec{\Sigma } \mathbf{B}+ \mathbf{B}\varvec{\Sigma }), \end{aligned}$$

in which we emphasize that here we also include the additional terms $2W_7 (\varvec{\Sigma } \mathbf{B}+\mathbf{B}\varvec{\Sigma })$ in (9).

We now simplify the strain-energy function further by using the empirical fact that shear components of the residual stress are negligible in the local coordinate system ($\mathbf{f}_0,\,\mathbf{s}_0$, and $\mathbf{n}_0$), as suggested by Costa’s measurements on canine LV (Costa et al. 1997), also shown below. Hence, the residual stress-related invariants $I_6$ and $I_7$ may be represented by the existing invariants in the Holzapfel and Ogden model (Holzapfel and Ogden 2009). This is because if the residual stress components $\tau _\text{ ff },\,\tau _\text{ ss }$, and $\tau _\text{ nn }$ are in the principal directions, then we can write

17 $$\begin{aligned} I_6=\text{ tr }(\varvec{\tau }\mathbf{C})=\tau _\text{ ss } I_{4\text{ s }}+\tau _\text{ ff }I_{4\text{ f }} +\tau _\text{ nn }I_{4\text{ n }}, \end{aligned}$$

in which $I_{4\text{ n }}$ is the $I_4$ invariant associated with the sheet–normal direction. We can also write

18 $$\begin{aligned} I_7=\text{ tr }(\varvec{\tau }\mathbf{C^2})=\tau _\text{ ss }I_{5\text{ s }} +\tau _\text{ ff }I_{5\text{ f }}+\tau _\text{ nn }I_{5\text{ n }}, \end{aligned}$$

in which $I_{\mathrm{5s}}=\mathbf{s}_0 \cdot (\mathbf{C}^2\mathbf{s}_0)$ and similarly for $I_{\mathrm{5f}}$ and $I_{\mathrm{5n}}.$ Since

19 $$\begin{aligned} I_1=\text{ tr }(\mathbf{C})=I_{4\text{ s }}+ I_{4\text{ f }}+I_{\mathrm{4n}}, \end{aligned}$$

we can also write

20 $$\begin{aligned} I_6=\tau _\text{ nn } I_1+(\tau _\text{ ff }-\tau _\text{ nn }) I_{4\text{ f }}+(\tau _\text{ ss }-\tau _\text{ nn })I_{4\text{ s }}. \end{aligned}$$

Hence, we can simply use a modified Holzapfel-Ogden model for a residually stressed myocardium. In the simpler approach, this is

21 $$\begin{aligned} W&= \frac{a}{2b}\exp [b(I_1-3)]\nonumber \\&\quad + \sum _{i=\text{ f },\text{ s }} {\frac{a_i}{2b_i}\{\exp [b_i(I_{4i}-1)^2]-1\}} \nonumber \\&\quad + \frac{a_\text{ fs }}{2b_\text{ fs }} \{\exp [b_\text{ fs }(I_{4\text{ fs }})^2]-1\} \nonumber \\&\quad + \frac{\tau _\text{ nn }}{2}I_1 + \frac{(\tau _\text{ ff }-\tau _\text{ nn })}{2}I_{4\text{ f }} + \frac{(\tau _\text{ ss }-\tau _\text{ nn })}{2}I_{4\text{ s }}, \end{aligned}$$

in which $\tau _{ii}$ for $i=\mathrm{f,s}$, and n are functions of $\mathbf{X} \in B_r$. The modified model for the extended approach can be similarly derived, in which $I_{5i}$ ($i=\mathrm{f,s}$, and n) may be written in terms of $I_{4i}$ and $I_{4ij}$ for $i,j=\mathrm{f,s}$, and n (Holzapfel and Ogden 2009).

In our computations, we choose the eight constitutive parameters to be those of Wang et al. (2013), who fit the parameters of the Holzapfel and Ogden (2009) model to the experimental data of Dokos et al. (2002). These parameters are shown in Table 1. The initial Cauchy stresses are determined presently.

**Table 1:** The material parameters from Wang et al. (2013)

$a$ (kPa)	$b$	$a_{\mathrm{f}}$ (kPa)	$b_{\mathrm{f}}$	$a_{\mathrm{s}}$ (kPa)	$b_{\mathrm{s}}$	$a_{\mathrm{fs}}$ (kPa)	$b_{\mathrm{fs}}$
0.236	10.81	20.04	14.15	3.72	5.16	0.41	11.3

### Estimate of the residual stress

It is important to note that the zero-stressed reference configuration $B_0$ defined here is fictitious, as the deformations inducing residual stresses are probably not compatible, and probably involve microscopic phenomena such as cell rotations. In particular, residual stresses likely arise from motions that cannot be described within the standard framework of continuum mechanics. With this in mind, we propose two methods to estimate the residual stress for the LV in $B_r$ with respect to $B_0$. (The method used to determine the unloaded configuration $B_r$ is described below in Sect. 2.5.) One approach to estimating the residual stress is to use the measured residual strain field of Costa et al. (1997), and the other is to use the opening angle method.

#### Residual stress estimated from data of Costa et al. (1997)

**Fig. 3:**
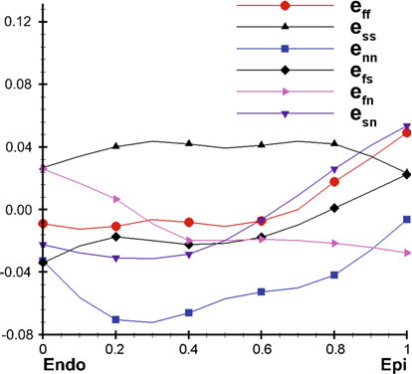
Three-dimensional residual strain distribution, replotted from the experimental measurements on canine LV by Costa et al. (1997)

Non-homogeneous three-dimensional residual strains in the mid-anterior part of canine left ventricle measured by Costa et al. (1997) are shown in Fig. 3. In the unloaded but residually stressed configuration $B_r$, the Euler–Almansi strain tensor (relative to the stress-free configuration $B_0$) is denoted by $\mathbf{e}_0$. If the deformation gradient from the stress-free configuration $B_0$ to the unloaded and residually stressed configuration $B_r$ is $\mathbf{F}_0$, and the deformation gradient from $B_r$ to $B_0$ is $\mathbf{F}^{-1}_0$, as shown in Fig. 1, then the left Cauchy–Green tensor, say $\mathbf{B}^{(r)}$, is

22 $$\begin{aligned} \mathbf{B}^{(r)} = \mathbf{F}_0\mathbf{F}_0^T=(\mathbb{I } - 2\mathbf{e}_0)^{-1}. \end{aligned}$$

Notice that $\mathbf{B}^{(r)}$ is generally different from $\mathbf{B}$. Specifically $\mathbf{B}=\mathbb{I }$ in $B_r$, but generally $\mathbf{B}^{(r)}\ne \mathbb{I }$.

We further assume that the fibres are relaxed in the unloaded but residually stressed configuration $B_r$. Indeed, there is experimental evidence that the collagen fibres are coiled and wavy in their unloaded state in arteries (Clark and Glagov 1985; Dingemans et al. 2000). By considering the deformation from $B_0$ to $B_r$, we calculate the residual stresses using the isotropic part of (3):

23 $$\begin{aligned} \hat{W}(I_1^{(r)}) = \frac{a}{2b}\exp [b(I_1^{(r)}-3)], \end{aligned}$$

in which $I_1^{(r)} = \text{ tr }(\mathbf{B}^{(r)})$. Then,

24 $$\begin{aligned} \varvec{\tau }&= \mathbf{F}_0 \frac{\partial \hat{W}}{\partial \mathbf{F}_0}(\mathbf{F}_0) -p^{(r)}\mathbb{I } \nonumber \\&= a\exp [b(I_1^{(r)}-3)]\mathbf{B}^{(r)}-p^{(r)}\mathbb{I } , \end{aligned}$$

with

25 $$\begin{aligned} p^{(r)}=2{W}_1=a \end{aligned}$$

from (12)$_1$. Using Eqs. (22)–(24), with the help of (25), we can completely determine the residual stress tensor.

Note the residual strain generated from this method is not compatible because the average strain components were used. In addition, the experimental data are only from along the mid-anterior free wall of the canine LV. The residual stress tensor thus constructed is only suited for a cylindrical section of the LV.

#### Residual stress estimated by the opening angle method

**Fig. 4:**
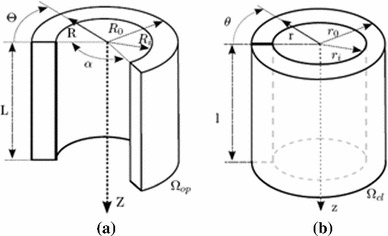
**a** The stress-free configuration after a radial cut, and **b** the unloaded cylindrical tube with residual stress (sketch taken from Alastrué et al. (2008))

Residual stress may be determined by the opening angle method (Guccione et al. 1991; Rachev and Greenwald 2003; Alastrué et al. 2008). Here, we approximate the LV as a cylindrical tube, $\varOmega _\text{ cl }$, and assume that the residual stress can be released by a radial cut and that the corresponding compatible stress-free configuration is an opened cylindrical sector $\varOmega _\text{ op }$ with opening angle $\alpha$, as shown in Fig. 4.

The opened cylinder is defined in the cylindrical coordinate system with unit vectors $({\mathbf{E}_R,\mathbf{E}_{\varTheta },\mathbf{E}_Z})$ as

26 $$\begin{aligned} R_\mathrm{i } \le R \le R_\mathrm{o },\,0 \le \varTheta \le (2\pi - \alpha ),\,0 \le Z \le L, \end{aligned}$$

in which $R_\text{ i },\,R_\text{ o }$, and $L$ denote the inner and outer radii and the length of the ring in the stress-free configuration, respectively. The closed cylinder is defined in the cylindrical coordinate system $({\mathbf{e}_r,\mathbf{e}_{\theta },\mathbf{e}_z})$ as

27 $$\begin{aligned} r_\mathrm{i } \le r \le r_\mathrm{o },\,0 \le \theta \le 2\pi ,\,0 \le z \le l, \end{aligned}$$

in which $r_\mathrm{i },\,r_\mathrm{o }$, and $l$ denote the inner and outer radii and the length of the ring, respectively, in the $\varOmega _\text{ cl }$ configuration. The expressions that determine the values for these parameters are

28 $$\begin{aligned} r\!=\!\sqrt{\frac{R^2\!-\!R_\mathrm{i }^2}{\kappa \lambda _z}\!+\!r_\mathrm{i }^2},\,\theta \!=\! \kappa \varTheta ,\,z \!=\! \lambda _z,\,\kappa \!=\! 2 \pi /(2\pi \!-\!\alpha ),\qquad \quad \end{aligned}$$

in which $\lambda _z$ is the axial stretch, which is assumed to be constant. The parameter $\kappa$ is a measure of the opening angle.

From the incompressibility constraint, we have that $\lambda _r \lambda _\theta \lambda _z = 1$, which allows us to express the other two principal stretches in cylindrical coordinates as functions of $\kappa$ and $r$,

29 $$\begin{aligned} \lambda _r(R)=\frac{\partial r}{\partial R}=\frac{R}{r\kappa \lambda _z},\,\lambda _\theta (R)=\frac{r}{R} \frac{\partial \theta }{\partial \varTheta } = \frac{\kappa r}{R}. \end{aligned}$$

Ignoring end effects, the only non-trivial equilibrium equation in the cylindrical coordinate system $({\mathbf{e}_r,\mathbf{e}_{\theta },\mathbf{e}_z})$ is

30 $$\begin{aligned} \frac{\text{ d }\tau _{rr}}{\text{ d }r}+\frac{\tau _{rr}- \tau _{\theta \theta }}{r}=0, \end{aligned}$$

with boundary conditions

31 $$\begin{aligned} \tau _{rr}=0, \text{ for } r=r_\text{ i } \text{ and } r=r_\text{ o }. \end{aligned}$$

Integrating (30) gives

32 $$\begin{aligned} \tau _{rr}=\int \limits _{r_\text{ o }}^{r} \frac{\tau _{\theta \theta } - \tau _{rr}}{r}\mathrm{d }r \end{aligned}$$

For given values of $\alpha ,\,R_\mathrm{i }$, $R_\mathrm{o }$, and $\lambda _z$, and with the help of (24), we can solve for the circumferential stress $\tau _{\theta \theta }$, radial stress $\tau _{rr}$, and longitudinal stress $\tau _{zz}$. Since the shear components are all zero, this gives us the residual stress tensor $\overline{\varvec{\tau }}$ in the cylindrical coordinate system $({\mathbf{e}_r,\mathbf{e}_{\theta },\mathbf{e}_z})$. The residual stress tensor $\varvec{\tau }$ in the fibre coordinate system $({\mathbf{f}_0,\mathbf{s}_0,\mathbf{n}_0})$ is obtained via

33 $$\begin{aligned} \varvec{\tau }= \mathbf{Q}^T\overline{\varvec{\tau }}\mathbf{Q}, \end{aligned}$$

in which $\mathbf{Q}$ is the rotation connecting the two coordinate systems. The shear components in the fibre coordinate system are small but non-zero. We assume that these are negligible, however, in view of the experimental data (Costa et al. 1997).

### The finite element model

The geometry of the human LV model is derived from MR imaging of a healthy volunteer (male, age 28) acquired at the end of diastole, which is identified by the peak of the R-wave from the subject’s ECG. The computational approach that we adopt to model the passive mechanics of the LV is based on the classical pressure-dilatation-displacement three-field  formulation commonly used to overcome locking problems exhibited by purely displacement-based finite element formulations of incompressible elasticity. We use the decoupled volumetric-isochoric formulation of finite elasticity and decompose the strain-energy function into volumetric and isochoric parts, with the incompressibility ensured using a Lagrange multiplier method. Detailed descriptions of the generation of the geometry and fibre structure, as well as the finite element procedure, were described previously (Wang et al. 2013). The LV model is discretized with 48,050 hexahedral elements and 53,548 nodes. Validations using different meshes and elements were performed in our previous work (Wang et al. 2013).

To constrain the motion of the model, the longitudinal displacement of the base and the circumferential displacement of the epicardial wall at the base are set to zero. The remainder of the left ventricular wall, including the apex, is left free. A pressure load, generally varying from 0 to 8 mmHg, is applied on the endocardial surface. Such loads are typical physiological end-diastolic pressures.

### The unloaded configuration

An essential requirement of the finite strain model is an unloaded reference geometry. Since the end-diastolic pressure is not zero, this requires the determination of unloaded reference geometries that are different from the imaged ones (Walker et al. 2005; Sermesant and Razavi 2010). For this purpose, we use an inverse displacement algorithm (Bols et al. 2011), which is especially easy to implement within a finite element framework. Other similar methods to determining the unloaded geometry include methods that compute a multiplicative decomposition of the deformation gradient tensor (Rodriguez et al. 1994; Aguado-Sierra et al. 2011). The objective of the iterative scheme is to find the unloaded reference geometry that minimizes the difference between the measured end-diastolic geometry and the computed geometry when the unloaded model is inflated to the measured end-diastolic pressures.

**Fig. 5:**
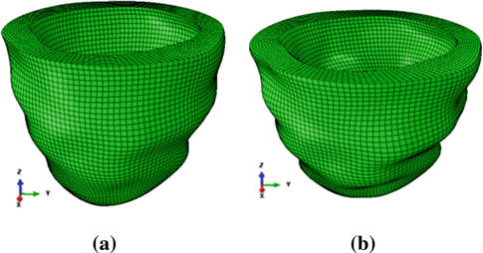
**a** The imaged end-diastole configuration at a loading pressure 8 mmHg, and **b** the unloaded configuration determined by an inverse displacement analysis

The basic procedure is as follows. In the first step, the imaging-derived end-diastolic model is treated as an unloaded reference state and inflated to an end-diastolic LV pressure. An approximation to the inverse of the resulting deformation is then applied to the imaged end-diastolic mesh using a backward-displacement method, thereby yielding a new unloaded reference configuration. This procedure is repeated until convergence is achieved. For further details, see Bols et al. (2011). This procedure converged to within $2.7\,\%$ of the measured chamber volumes in six iterations. The maximum displacement between the computed and measured end-diastolic geometries is about $0.15~\text{ mm }$.   The finite element meshes based on MR images at the end-diastolic and estimated unloaded configurations are shown in Fig. 5. Because it is difficult to keep track of the fibre structure during these iterations, a new rule-based fibre structure is generated for the finite element mesh in each newly generated reference configuration.

The parameters are chosen to be:  $\alpha =85^\circ$, along with $R_i=2.5~\text{ cm },\,R_o=4.2~\text{ cm }$, and $\lambda _z=1$.

### Finite strain in the LV model with residual stress

**Fig. 6:**
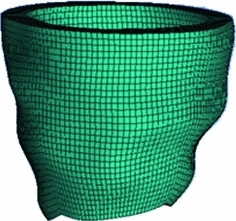
The “cylindrical region” of the LV, where residual stress is considered. Residual stresses are not included near the apex

Because the residual stress tensor is estimated from limited experimental data or from the opening angle method for a cylinder, we are only able to consider the residual stress in the “cylindrical region” of the LV geometry shown in Fig. 6. To compute the total stress under external loading, we choose the rule-based fibre structure with the fibre angle changing from $-60^\circ$ to $+60^\circ$, and the sheet angle from $-45^\circ$ to $+45^\circ$, as used by Wang et al. (2013). We consider two loading pressures: 3 mmHg (early diastole) and 8 mmHg (late diastole).

## Results

### Residual stress estimates

#### Residual stress from Costa’s residual strain measurements

**Fig. 7:**
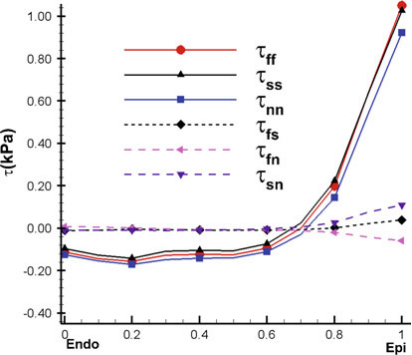
The transmural residual stress along the mid-anterior free wall of the LV model at approximately two-third the distance from base to apex, using Costa’s residual strain data

Using (23) and (24), we can compute the transmural distributions of the residual stress along the mid-anterior free wall at approximately two-thirds the distance from base to apex. Figure 7 shows that the residual stress $\tau _\text{ ff }$ is slightly compressive on the endocardial surface and tensile near the epicardial surface. This agrees with the result for residual stresses in arteries (Liu and Fung 1989; Han and Fung 1996). The overall magnitude of the residual stress components is not large, which has also been observed previously (Guccione et al. 1991; Nash 1998). In particular, we observe that all the shear components are negligible, and hence $\tau _\text{ ff },\,\tau _\text{ ss }$, and $\tau _\text{ nn }$ can be considered as the principal residual stresses.

#### Residual stresses by the opening angle method

**Fig. 8:**
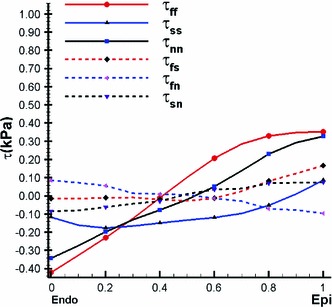
Transmural distributions of the residual stress estimated using the opening angle method in the unloaded configuration for an opening angle of $85^\circ$

For comparison, we use an opening angle $\alpha =85^\circ$ in the following computations. The transmural distributions of the residual stress components are shown in Fig. 8. Results from both the opening angle method and the Costa measurements are in good qualitative agreement with the results of previous research (Guccione et al. 1991; Taber 1991) in that there is an increased residual stress that is tensile near the sub-epicardial wall, and increased compression near the endocardium. This residually stressed configuration helps the LV wall to cope with the physiological pressure loading. However, the opening angle method seems to underestimate significantly the residual stress in the LV; it has been reported that the mean opening angle in the rat LV is about $45^\circ$ (Omens and Fung 1990), whereas we found the opening angle method can only predict a residual strain comparable in magnitude to the measurement by Costa et al. (1997) when the opening angle is increased to $85^\circ$. This suggests that the residual stress within the LV may not be released by a single cut and that 3D measurements are necessary to provide accurate estimate for residual strains. We also observe that the shear components are non-zero when using the opening angle method, but these are much smaller than the normal components as shown in Fig. 8 and hence can be neglected.

#### Early diastole

**Fig. 9:**
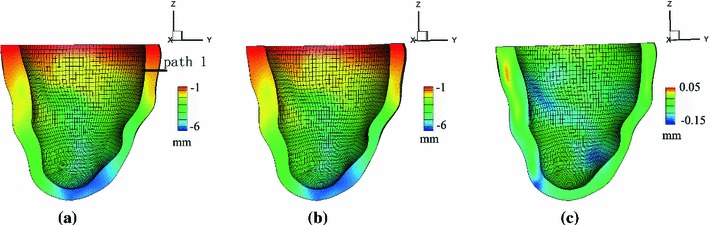
The three-dimensional distribution of the vertical displacement in the LV for the model with residual stress (**a**), without residual stress (**b**), and their difference (**c**). The simpler approach to incorporating residual stresses is used to obtain these results. Notice that the path over which stresses are displayed in subsequent figures is shown in panel (**a**)

**Fig. 10:**
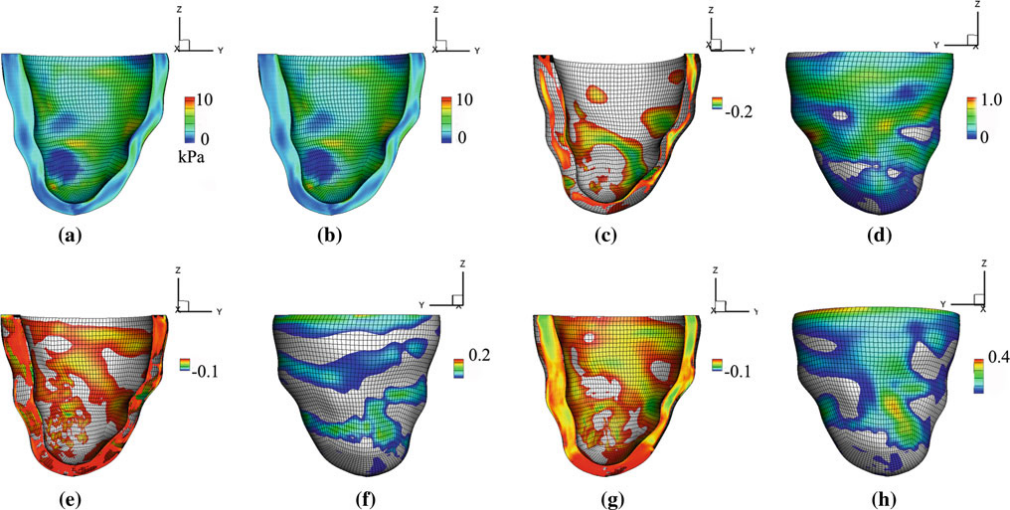
The fibre stress $\sigma _\text{ ff }$ for the model with residual stress from the Costa data (**a**); similar to (**a**), but here without residual stress (**b**); the region in which the difference between (**a**) and (**b**) is negative (**c**); similar to (**c**), but here showing the region where the difference is positive (**d**); the region where the difference between $\sigma _\text{ ss }$ with and without residual stress is negative (**e**); similar to (**e**), but here showing the region where the difference is positive (**f**); the region where the difference between $\sigma _\text{ nn }$ with and without residual stress is negative (**g**); and similar to (**g**), but here showing the region where the difference is positive (**h**). The simpler approach to incorporating residual stresses is used to obtain these results

Using data from Costa et al. (1997), we show the vertical displacement with and without the residual stress and their difference in Fig. 9. The average difference is $-0.05 \pm 0.05~\text{ mm }$, indicating the effect of the residual stress is to shorten the long axis slightly. The three-dimensional distributions of the fibre stress $\sigma _\text{ ff }$ in the LV from the model with and without residual stress are shown in Fig. 10. Notice that the distributions obtained with and without residual stresses are similar. Notice also that Fig. 10d shows that for most of the epicardial region, including residual stresses increases $\sigma _\text{ ff }$.

**Fig. 11:**
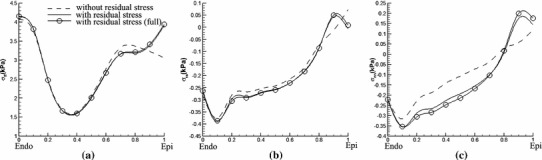
Transmural distribution of Cauchy stress components along path 1 shown in Fig. 9a for **a** $\sigma _\text{ ff }$, **b** $\sigma _\text{ ss }$, and **c** $\sigma _\text{ nn }$. *Solid curves* indicate the total stress with the residual stress estimated from the data of Costa et al. (1997) using the simple approach to incorporating residual strains, using the extended approach (*marked with circles*), and without residual stress (*dashed*). In these simulations, the endocardial loading pressure is $3$ mmHg

The transmural distribution of the fibre stress along one mid-ventricular path is shown in Fig. 11; the path is shown in Fig. 9a. The residual stress mainly affects $\sigma _\text{ ff }$ in the sub-epicardial region ($28\,\%$ increase at the epicardial surface), whereas for $\sigma _\text{ ss }$ and $\sigma _\text{ nn }$, the effect of residual stress is to increase the stresses in the sub-epicardial region and to decrease the stresses in the sub-endocardial region.

**Fig. 12:**
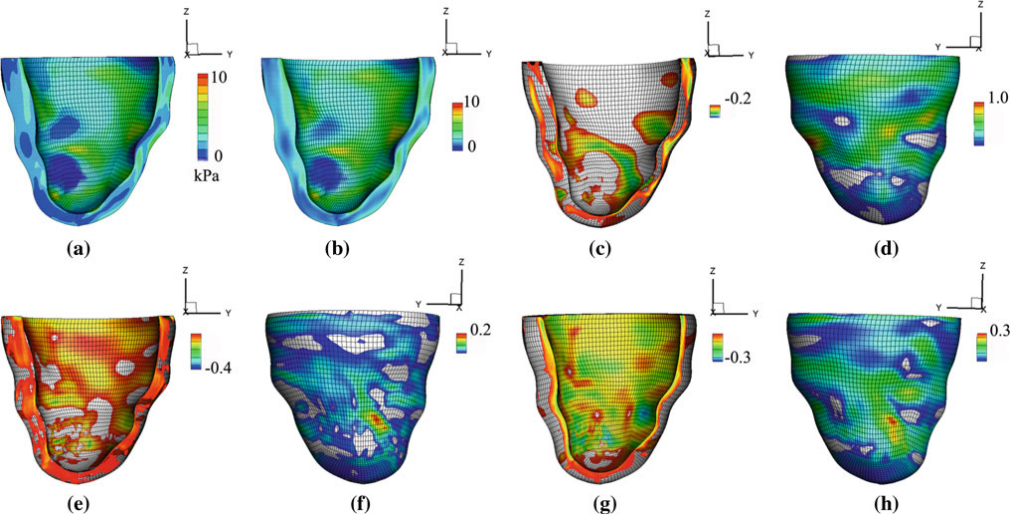
Similar to Fig. 10, but here using the opening angle method to determine the residual stresses

**Fig. 13:**
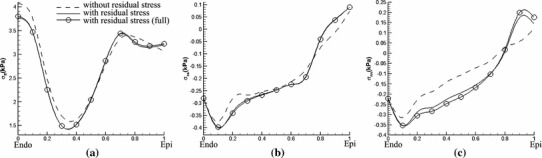
Similar to Fig. 11, but here using the opening angle method to determine the residual stresses

Results obtained by the opening angle method are similar and are shown in Fig. 12. The transmural stress distribution along the mid-ventricle path is shown in Fig. 13.

Table 2 summarizes the mean stress components $\sigma _\text{ ff },\,\sigma _\text{ ss }$, and $\sigma _\text{ nn }$ at the endocardial and epicardial surfaces with and without the residual stresses. To help to interpret these results, the differences of these stress components obtained with and without including residual stresses are also shown. It is more evident from the opening angle method that the residual stress seems to increase stress in the epicardial surface and decrease it in the endocardial surface. The averages of $\sigma _\text{ ff },\,\sigma _\text{ ss }$, and $\sigma _\text{ nn }$ are decreased by 6 %,   6 %, and 27 % along the endocardial surface, respectively, and increased by $7$ %, $50$ %, and $45\,\%$ along the epicardial surface, respectively.

**Table 2:** Stress means and standard deviations along the endocardial and epicardial surfaces at an endocardial loading pressure of $3$ mmHg

	$\sigma _\text{ ff }$ (kPa)	$\sigma _\text{ ss }$ (kPa)	$\sigma _\text{ nn }$ (kPa)
*Endocardial surface*			
Without $\varvec{\tau }$	$2.9\pm 1.6$	$-0.33\pm 0.32$	$-0.15 \pm 0.35$
With $\varvec{\tau }$ (Costa et al. data)	$2.9\pm 1.6$	$-0.34\pm 0.31$	$-0.17 \pm 0.35$
With $\varvec{\tau }$ (opening angle)	$2.8\pm 1.5$	$-0.35\pm 0.30$	$-0.20 \pm 0.35$
Difference (Costa et al. data)	$-0.01\pm 0.07$	$-0.004\pm 0.01$	$-0.01 \pm 0.02$
Difference (opening angle)	$-0.16\pm 0.15$	$-0.02\pm 0.05$	$-0.04 \pm 0.06$
*Epicardial surface*			
Without $\varvec{\tau }$	$1.23 \pm 0.77$	$0.04 \pm 0.08$	$0.11 \pm 0.13$
With $\varvec{\tau }$ (Costa et al. data)	$1.28 \pm 0.8$	$0.04 \pm 0.08$	$0.11 \pm 0.13$
With $\varvec{\tau }$ (opening angle)	$1.31 \pm 0.8$	$0.06 \pm 0.08$	$0.16 \pm 0.14$
Difference (Costa et al. data)	$0.05\pm 0.09$	$0.0\pm 0.02$	$0.0\pm 0.02$
Difference (opening angle)	$0.08\pm 0.11$	$0.02\pm 0.03$	$0.05 \pm 0.04$

Both the direct strain measurement and the opening angle method show that there are some non-negligible differences in the stress distribution when residual stress is included, particularly near the epicardial region. As discovered by Guccione et al. (1991), Taber (1991), Nash (1998), and others, residual stress tends to reduce the total stress in the sub-endocardium and to increase the stress in the sub-epicardium, therefore releasing the stretch of SL during loading. This is particularly evident for the residual stress resulting from the opening angle method, as in Fig. 12. The results also demonstrate that both methods of estimating the residual stress give quantitatively similar results.

To see whether the simpler approach (i.e. considering only the $I_6$ term in the strain-energy function with residual stresses) is sufficiently accurate for these analyses, we also compare the results obtained by the simple approach to results obtained by the extended approach (i.e. in which the effects of the $I_7$ term are also included). Comparisons between the two approaches are shown in Figs. 11 and 13. It is clear that the results from both approaches are almost identical. We conclude that the simpler model is sufficient for this case.

#### Late diastole

**Fig. 14:**
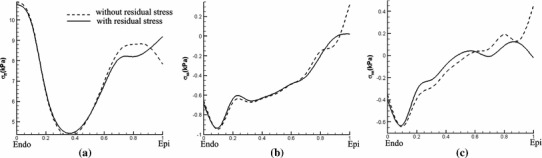
Transmural distributions of the Cauchy stress components for **a** $\sigma _\text{ ff }$, **b** $\sigma _\text{ ss }$, and **c** $\sigma _\text{ nn }$ along the mid-ventricular path at an endocardial pressure of 8 mmHg. The *solid curves* show the total stress including the residual stress estimated from the Costa data, and the *dashed curves* exclude the residual stress. Only the simpler approach is used

**Fig. 15:**
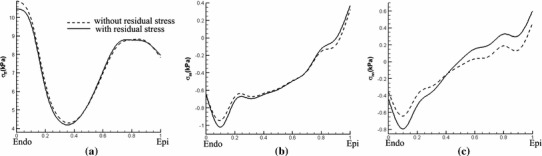
Similar to Fig. 14, but here using the opening angle method to determine the residual stresses

The transmural distribution of the stress along the mid-ventricular path obtained when the endocardial loading pressure is increased to 8 mmHg is shown in Fig. 14 for residual stresses obtained from the Costa data and in Fig. 15 for residual stresses obtained by the opening angle method. These results show that the overall impact of the residual stress is similar to that of early diastolic pressure loads, but in this case, the net effect of the residual stresses is reduced. Differences between the results from the Costa data and the opening angle method are somewhat greater in late diastole as compared to early diastole. However, because both methods involve significant simplifications for the LV model, we do not further discuss these differences. These results are included mainly to demonstrate that including residual stress could change the total stress in the physiologically loaded state.

#### Pressure–volume relations

**Fig. 16:**
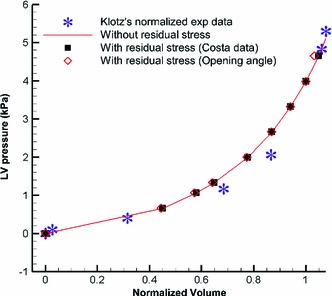
The normalized pressure–volume curves with and without residual stresses. All of these agree well with the experimental measurements by Klotz et al. (2006)

Finally, we determine the end-diastolic pressure–volume relation with and without the residual stresses. Results are shown in Fig. 16. We find that the pressure–volume curve seems not to be affected by the presence of the residual stress. This agrees with the modelling prediction of Taber (1991), as well as measurements of Klotz et al. (2006), who also confirmed that the influence of the residual stress on the pressure–volume curve is negligible. This is not surprising, because classical measurements like *P*–*V* loops are often less sensitive to detailed mechanical changes. A similar finding was reported by Eriksson et al. (2012), who found that the non-negligible role of heterogeneity in structural fibre/sheet orthotropy is not reflected in the *P*–*V* curves generated by models with or without heterogeneity.

## Discussion

We have modelled the effects of residual stresses on LV mechanics using a modified Holzapfel-Ogden model. Applications of this model show that the residual stress in the human LV could make non-negligible changes in the stress distributions at physiological pressures in diastole. Since estimates of the residual stress are necessarily limited by the lack of three-dimensional strain data in the LV, the results presented herein are therefore primarily illustrative, and may not give the full scale of the impact of residual stress in the LV. With the given assumptions, however, we have not found any changes in the pressure–volume curve in diastole when incorporating residual stresses into our model.

**Fig. 17:**
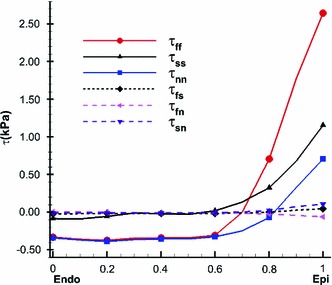
Transmural distributions of the residual stress in the unloaded configuration, in which the residual stress is estimated from the strain measured by Costa et al. (1997) and the full orthotropic Holzapfel-Ogden model

**Fig. 18:**
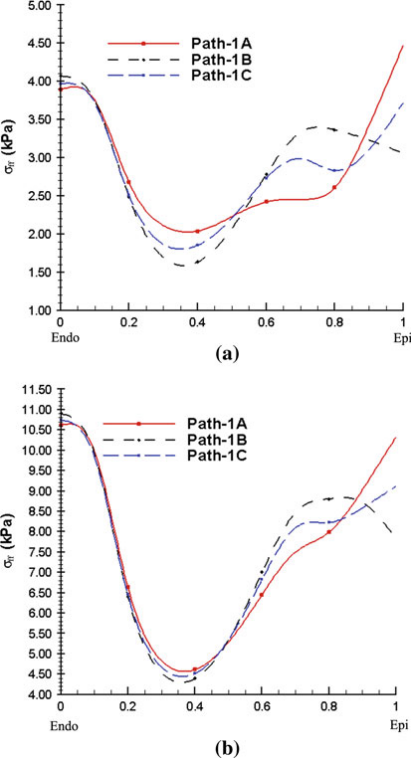
The transmural fibre stress distribution at an endocardial pressure load of $3$ mmHg (**a**) and $8$ mmHg (**b**). Here, *A* is the curve with residual stress estimated from the anisotropic model, *B* is without the residual stress, and *C* is with the isotropic model. In all computations, the simpler approach is used

In the foregoing, we have assumed that the residual stress behavior is governed by the isotropic matrix, which may be a point of debate. To evaluate the impact of this assumption on the determined residual strains and stress distributions upon endocardial pressure loading, we also perform similar computations, but now employing the full orthotropic Holzapfel-Ogden model to evaluate the residual stress. Results are shown in Figs. 17 and 18. These data show that the sub-epicardial fibre stress is even greater if we use the full anisotropic model. Hence, the impact of using a different constitutive model for residual stress could be significant. This poses further research challenges concerning the choice of the most suitable constitutive law for evaluating the residual stress, probably at the microscopic level. However, we note that, as before, the pressure–volume curve is not affected by the residual stress if we use the anisotropic model (data not shown).

In determining the unloaded configuration, we have made two further assumptions. One is that the unloaded configuration is in equilibrium with the addition of the residual stresses obtained from limited experimental data or by simplified opening angle methods. In practice, the residual stresses computed by either approach do not strictly satisfy the condition (5), and hence the addition of residual strains results in a shift in the unloaded configuration. It may be important to determine the unloaded configuration and the residual stress distributions simultaneously; however, doing so is beyond the scope of the present work. In addition, because we have access only to limited experimental data on residual stresses or strains, it is not clear that the significant additional effort required to determine the unloaded configuration and residual stress states simultaneously is well justified. The other assumption is that the fibre structure may be regenerated using the rule-based algorithm in the unloaded configuration. This last assumption is made because it is difficult to track the fibre structure during the inverse analysis; regenerating the fibre structure simplifies the implementation significantly.

In an effort to find the simplest way to include the residual stresses, we have shown that residual stress in the myocardium can be represented accurately by a modified Holzapfel-Ogden model. This modified model can readily be applied to similar problems in which the residual stress tensor has the principal directions in the fibre coordinate system. For more general problems, our results suggest that the contribution from a single $I_6$ term is sufficient to model the residual stress.

Overall, the residual stress has some effects, particularly on the fibre stress and cross-fibre stress, although the extent of this seems to be less significant later in diastole when pressure is higher. However, it does not necessaily follow that the impact of the residual stress would be further decreased in systole, when the pressure reaches its peak. This is because the additional coupling terms of residual stress to larger strains and fibre directions will come into play, which are ignored in the present study. When a more general theory similar to that of Ogden and Singh (2011) is employed, the effect of the residul stress  on the whole cardiac cycle may be more significant.This may also be the case when more detailed experimental measurements of residual strain are available for the whole LV, including the apex. In addition, we remark that the presence of residual stress may have a significant impact on myocardial growth and remodelling, which have not been modelled here. Indeed, the growth and remodelling processes may themselves act to establish and to maintain residual stresses.

## Conclusions

In this paper, we proposed a modified Holzapfel-Ogden model for the myocardium in diastole which includes a basic contribution from residual stress. This work follows on the theoretical framework of Holzapfel and Ogden (2009) and of Shams et al. (2011). We found that using only one extra invariant, $I_6$, in the strain-energy function is sufficiently accurate for the finite strain models considered herein. The modified constitutive model allows the residual stress to be considered without too much additional computational effort. Using this constitutive law, we carried out stress and strain analyses using a MRI-derived human left ventricular model. The residual stress is applied to the unloaded configuration, which is determined by an inverse displacement analysis. Two different methods of estimating the residual strain are considered. One is from the three-dimensional strain measurements of canine LV, and the other uses the opening angle method. Each of these methods involves simplifications and can only estimate the residual stress in the cylindrical section of the LV, which could underestimates the magnitude of the true residual stress field inside the LV. Our results show that even the simplified residual stress representations, in which the coupling terms with fibre directions are ignored, could make non-negligible changes in the stress distribution in the human LV model at physiological pressures in diastole. Further experimental data are required to investigate fully the effects of residual stresses and to determine the most suitable constitutive laws for capturing the residual strain and stress in the LV.

## Footnotes

1 We use the terms initial stress and residual stress interchangeably, which is in line with the definition used by Biot and Romain (1965).
